# Sources of Hepatitis E Virus Genotype 3 in the Netherlands

**DOI:** 10.3201/eid1503.071472

**Published:** 2009-03

**Authors:** Saskia A. Rutjes, Willemijn J. Lodder, Froukje Lodder-Verschoor, Harold H.J.L. van den Berg, Harry Vennema, Erwin Duizer, Marion Koopmans, Ana Maria de Roda Husman

**Affiliations:** National Institute for Public Health and the Environment, Bilthoven, the Netherlands

**Keywords:** Hepatitis E virus, transmission route, reservoir, environment, human, research

## Abstract

Non–travel-related hepatitis E virus (HEV) genotype 3 infections in persons in the Netherlands may have a zoonotic, foodborne, or water-borne origin. Possible reservoirs for HEV transmission by water, food, and animals were studied. HEV genotype 3/open reading frame 2 sequences were detected in 53% of pig farms, 4% of wild boar feces, and 17% of surface water samples. HEV sequences grouped within 4 genotype 3 clusters, of which 1 is so far unique to the Netherlands. The 2 largest clusters contained 35% and 43% of the animal and environmental sequences and 75% and 6%, respectively, of human HEV sequences obtained from a study on Dutch hepatitis E patients. This finding suggests that infection risk may be also dependent on transmission routes other than the ones currently studied. Besides the route of exposure, virus characteristics may be an important determinant for HEV disease in humans.

## Abstract 2

Four subtypes have been detected, and pathogenicity, zoonotic potential, or stability may vary between subtypes.

Hepatitis E virus (HEV) is an RNA virus that causes liver inflammation in humans, predominantly in developing countries. In the 1990s, serologic studies among blood donors in industrialized countries showed that anti-HEV seropositivity also occurred among a small percentage (1.1%–1.4%) of persons without a travel history to a hepatitis E–endemic region (*1*,*2*). Later studies confirmed sporadic hepatitis E cases contracted in Europe, the United States, and other industrialized regions (*3*). HEV strains detected in mammals can be classified into 4 major genotypes that are represented by Burmese isolates (genotype 1), Mexican isolates (genotype 2), US isolates (genotype 3), and recent Chinese isolates (genotype 4) (*3*). In addition, virulent and avirulent HEV strains that infect birds have recently been identified (*4*,*5*). In industrialized countries, non–travel-related HEV infections are caused by genotype 3 (Europe, United States, Japan, New Zealand, Argentina) and genotype 4 (Japan, People’s Republic of China).

A possible role for zoonotic transmission in the epidemiology of human HEV episodes has been suggested after viral RNA was detected in different animal species, and these viruses were found to be closely related to HEV strains found in humans. The first animal in which HEV genotype 3 was identified and characterized was pig in the United States (*6*). HEV strains of genotypes 3 and 4 have since been detected in pigs in many other countries, and these strains were found to be genetically closely related to HEV strains originating from humans in the same geographic region (*7*,*8*). Serologic studies have also indicated a broad host range of HEV. In many animal species such as cows, cats, dogs, rodents, and mongooses, immunoglobulin G to HEV was detected by using several serologic tests. However, HEV RNA was not detected in these animals and because of the lack of positive reference materials to evaluate these tests, the results must be interpreted with caution (*9*–*12*).

Assuming a zoonotic source for HEV infections, exposure to reservoirs of HEV might occur through contact with infected animals and animal products. Consumption of contaminated food or drinking water or contact with contaminated surface waters may also expose humans to HEV. In Japan, identical fragments of HEV were obtained from strains isolated from deer, wild boar meat, and patients with hepatitis E who had consumed this meat (*13*–*15*).

In the Netherlands, the first report of non–travel-related HEV infections was published in 2003 (*16*). Since then, ≈10 cases/year of non–travel-related HEV infections have been diagnosed (*17*,*18*). In 2004–2006, a descriptive case study was performed to generate hypotheses about possible risk factors and transmission routes for non–travel-related HEV infections in the Netherlands (*19*). However, no conclusive evidence for a specific transmission route was observed. Therefore, we studied possible reservoirs for HEV transmission by water, food, and animals in the Netherlands. To assess possible differential risks of exposure, we compared these environmental sequences with sequences obtained from Dutch HEV patients whose conditions had been diagnosed in the same period.

## Materials and Methods

### Fecal Samples

Pooled fecal samples were collected from 97 pig farms located throughout the Netherlands (20–60 fresh stool specimens per farm) throughout 2005 (*20*). Individual fecal samples from 50 pigs (5 pigs from each of 10 farms) were collected at a slaughterhouse in the southern part of the Netherlands in November 2006. Fecal samples were collected from 150 muskrats (*Ondatra zibethicus*) that were caught in 2 regions in the Netherlands, in the northeast (Groningen) and in the southeast (Limburg) (*21*). Individual fecal samples were collected from 26 wild boars in National Park De Hoge Veluwe in the center region of the Netherlands (Gelderland) in 2005. Two fecal samples obtained from 2 pigs on 1 farm in 2002 were included in this study. Fecal samples were stored as 50% suspensions in 15 g/L of Trypton Soya broth (CM 129; Oxoid, Cambridge, UK) and 10% glycerol at –70°C until testing.

### Water Samples

From September 2004 through July 2005, twelve large-volume water samples (230 L–260 L) were collected from the Meuse River monthly by using a conventional filter adsorption–elution method and concentrated by ultrafiltration by using a cellulose-acetate filter (nominal molecular weight limit of 10,000) under high pressure (3 bar) (*22*). Resulting concentrates were stored at –70°C.

### Extraction of RNA

RNA was extracted from 100 μL of 10% pooled fecal suspensions from pigs or 0.1% fecal suspensions from muskrats according to the method Boom et al. (*23*). The QIAamp viral RNA mini kit (QIAGEN, Venlo, the Netherlands) was used to extract RNA from 140 μL of 10% fecal suspensions from individually sampled slaughterhouse pigs and 1% fecal suspensions from wild boars as described by the manufacturer. RNA was isolated from 12.5 μL of ultrafiltered water concentrate as described (*22*).

### Reverse Transcription–PCR for HEV

Reverse transcription–PCR (RT-PCR) was performed on 10-fold serially diluted RNA samples with primers HEVORF2con-s1 and HEVORF2con-a1, which were specific for the conserved open reading frame (ORF) 2 region, as described (*24*,*25*). An internal control RNA was included in the RT step to monitor for inhibition of the RT-PCR (*20*). A nested RT-PCR was used to detect ORF1 sequences coding for nonstructural proteins by using primers HEVConsORF1-s1 and HEVConsORF1-a1 (*24*) for the first round of amplification and primers ConsORF1-s2 and ConsORF1-a2 (*26*) for the second round as described (*25*). Negative controls were used, and measures were taken to prevent contamination by complete separation of activities for first- and second-round PCRs in time and space. Animal and environmental samples were analyzed in the Laboratory for Zoonoses and Environmental Microbiology of the National Institute for Public Health and the Environment (RIVM) in Bilthoven. Human sequences were obtained from the Laboratory for Infectious Diseases and Perinatal Screening of RIVM. Use of 2 physically separated laboratories excluded possible cross-contamination of human, animal, and environmental samples. Numbers of viral genomes in samples by RT-PCR (PCR-detectable units [PDUs]) were estimated as most probable numbers as described (*27*).

### Sequencing and Phylogenetic Analysis

HEV RT-PCR products positive by hybridization (ORF2) or of the correct size (ORF1) were subjected to electrophoresis on a 2% agarose gel, excised, purified by using a Qiaquick Gel Extraction Kit (QIAGEN, Hilden, Germany), and sequenced by using the BigDye Terminator Cycle Sequencing Ready Reaction Kit (Applied Biosystems, Nieuwerkerk a/d IJssel, the Netherlands). HEV RT-PCR products for which no sequences were obtained by direct sequencing were cloned into a pCRII-TOPO vector (Invitrogen, Breda, the Netherlands); >5 clones per product were sequenced. Nucleotide sequences without primer sequences were aligned and clustered by maximum-parsimony analysis in BioNumerics version 4.6 (Applied Maths, Kortrijk, Belgium) and corresponded with analysis of a 242-nt fragment of ORF1 and a 148-nt fragment of ORF2. Human HEV sequences were obtained from human serum samples used for diagnosis of acute viral hepatitis (*18*,*19*).

## Results

### Survey of Possible Sources of HEV

Ten-fold serially diluted RNA samples extracted from animal samples and river water were analyzed for HEV ORF2 sequences by RT-PCR. The highest prevalence of HEV ORF2 RNA in 51 (53%) of 97 pooled fecal samples was detected in pig farms housing pigs 5–27 weeks of age (Table 1). A prevalence of HEV RNA of 14% was detected in feces of pigs ≈6 months of age and ready for slaughter, which corresponded with a prevalence of 30% on pig farms. HEV RNA was detected in 1 (4%) of 26 wild boar fecal samples. Muskrat fecal samples contained many inhibitory factors for RT-PCR. Therefore, samples were tested at several dilutions (10%–0.1% vol/vol feces). HEV RNA was not detected in any of the muskrat fecal samples, although the possibility that some of the samples had false-negative results cannot be excluded because in 24 fecal samples no internal control RNA was detected.

**Table 1:** Detection of hepatitis E virus RNA by RT-PCR for the ORF2 region of the genome in environmental samples, the Netherlands*

Source	Origin	Sample type	Sampling year	Matrix	No. samples	ORF2 (nt 6298–6494)†	
						No. (%) PCR positive	Sequence
Pig	Pig farm	Pooled	2005	Feces	97	51 (53)	36‡
		Individual	2002	Feces	2	2 (100)	2
	Slaughterhouse	Individual	2006	Feces	50	7 (14)	7
	Butcher shop/ supermarket§	Individual	2005	Liver	62	4 (6)	3
Wild boar	National Park	Individual	2005	Feces	26	1 (4)	1
Muskrat	Southeast	Individual	1998–1999	Feces	150	0	0
Water	Meuse River	Filtered	2004–2005	Concentrate	12	2 (17)	2

*RT-PCR, reverse transcription–PCR; ORF, open reading frame. †Position in Burmese hepatitis E virus strain (GenBank accession no. M73218). ‡Obtained by direct sequencing. §Obtained from Bouwknegt et al. (*28*).

HEV RNA concentrations in positive fecal samples varied from 10^3^ to 10^6^ PDU/g. In 2 (17%) of 12 river water samples analyzed, HEV RNA was detected at concentrations of 2 PDU/L to 100 PDU/L.

### Phylogenetic Analyses of HEV in Animal and Environmental Samples (2004–2006)

Thirty-six newly generated nucleotide sequences of 148 nt of HEV ORF2 were obtained from pooled fecal samples from pig farms that were previously shown to be positive for HEV ORF1 sequences (*20*). Nine nucleotide sequences of HEV ORF2 were obtained from individually sampled pigs either on a pig farm or during slaughter. The latter samples represented the infectious status of pigs at the time of consumption. One HEV ORF2 sequence was obtained from an HEV-positive wild boar fecal sample and 2 from HEV-positive water samples (Table 1). These sequences, together with 3 previously published HEV sequences detected in Dutch pig livers (*28*), were compared with available genotype 3 HEV strains from animals and humans (www.ncbi.nlm.nih.gov) by phylogenetic analysis. This comparison showed that all belonged to HEV genotype 3.

**Figure 1:**
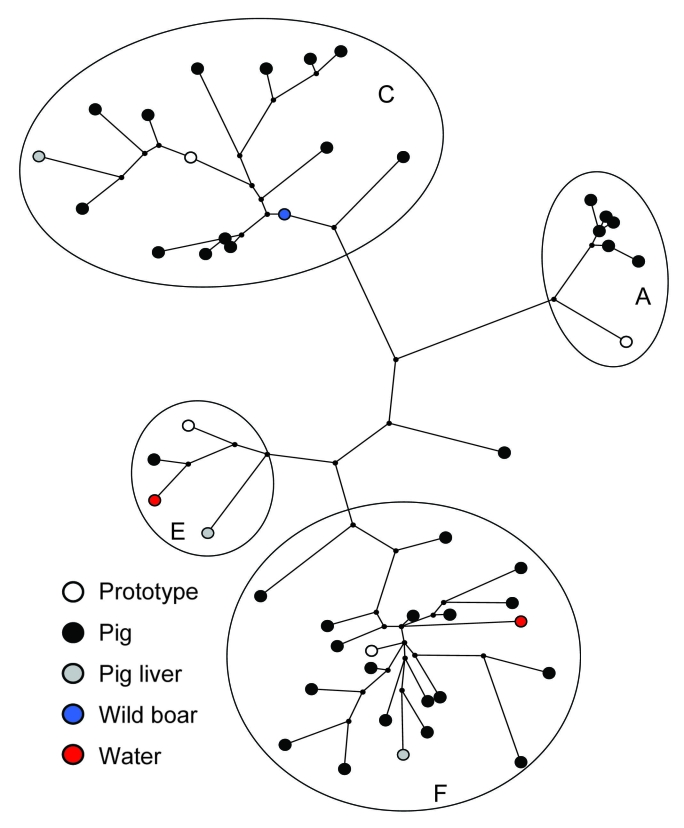
Maximum-parsimony tree of hepatitis E virus (HEV) sequences detected in pig, wild boar and water samples, based on a 148-nt sequence of open reading frame 2 (nt 6322–6469 of strain M73218). Sources of Dutch sequences and genotype 3 clusters are indicated. Sequences are compared with prototype sequences of different clusters of HEV genotype 3. Prototypes correspond with the following GenBank accession nos.: A) US1, AF060668; C) NLSW105, AF336298; E) UK-swine p354, AF503511; F) G1, AF110391. The following accession numbers have been used for phylogenetic analysis of isolates from surface water: EU526620, EU526626, from wild boar; EU526642, from pig liver; DQ916142–DQ916144, from pig feces; and DQ996399, EU526606–EU526619, EU526621–EU526625, EU526627–EU526641, EU526643–EU526647.

Sequences grouped within 4 previously proposed genotype 3 clusters (3a, 3c, 3e, and 3f) (*3*) (Figure 1), of which cluster 3c is unique to the Netherlands. Comparison of newly generated HEV ORF2 sequences obtained from pooled fecal pig samples with the previously published HEV ORF1 sequences from the same samples showed identical clustering in 4 HEV genotype 3 clusters (*20*). HEV genotype 3e sequences were not detected in the Netherlands until 2005. At 1 pig farm sampled in 2002, an unrelated HEV genotype 3 variant was detected. Most HEV sequences obtained from animals and water detected during 2004–2006 grouped within cluster 3f (43%): 18 HEV sequences isolated from pig farms, 1 from pig liver, and 1 from water, with identities of 87.2% to 97.3% (Table 2). The second largest cluster (3c) included 35% of environmental HEV sequences and contained 14 HEV sequences obtained from pig farms, 1 HEV sequence from pig liver, and 1 HEV sequence from wild boar. The identities ranged from 88.5% to 100%. Six sequences obtained from pig farms grouped within clusters 3a, with similarities ranging from 96.0% to 99.3%. Three HEV sequences obtained from pig liver, feces, and water grouped within cluster 3e. Analysis of the geographic distribution of all Dutch environmental HEV sequences by postal code showed that HEV sequences belonging to the 2 major clusters (3c and 3f) were distributed randomly. Too few HEV strains within clusters 3a and 3e were available to be informative for geographic distribution.

**Table 2:** Nucleotide identities between environmental and human HEV strains that circulated during 2004–2006, the Netherlands*

Cluster	Nucleotide identities between environmental HEV strains†				Nucleotide identities between human and environmental HEV strains†				Nucleotide identities between human HEV strains†		
	No. strains compared	Minimal, %	Maximal, %		No. strains compared	Minimal, %	Maximal, %		No. strains compared	Minimal, %	Maximal, %
3a	6	96.0	99.3		7	96.6	99.3		1	–	–
3c	16	88.5	100		28	87.2	100		12	88.2	100
3e	3	91.2	96.6		5	92.6	97.3		2	93.3	97.3
3f	20	87.2	97.3		21	88.5	93.9		1	–	–

*HEV, hepatitis E virus; –, no identities (1 human HEV sequence was present). †Based on a 148-nt sequence of open reading frame 2 (nt 6322–6469 of strain M73218).

### Phylogenetic Analyses of HEV ORF1 Sequences

Two sequences in cluster 3f obtained from different pig farms were 100% identical within the 148-nt fragment of ORF2. A total of 242 nt of ORF1 were sequenced and showed that the 2 sequences were 98.8% identical within this region of the HEV genome. This result indicated that the strains were similar but not identical.

### Comparison of Environmental HEV Sequences with Human HEV Sequences

**Figure 2:**
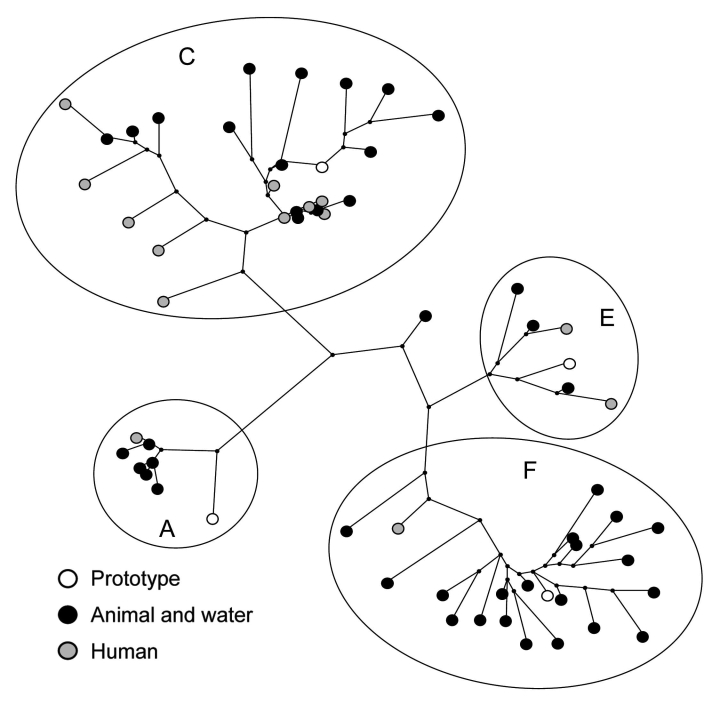
Maximum-parsimony tree of hepatitis E virus (HEV) sequences detected in environmental samples and patients during 2004–2006, based on a 148-nt sequence of open reading frame 2 (nt 6322–6469 of strain M73218). Origins of HEV sequences and genotype 3 clusters are indicated. Sequences are compared with prototype sequences of different clusters of HEV genotype 3. Prototypes correspond with the following GenBank accession nos.: A) US1, AF060668; C) NLSW105, AF336298; E) UK-swine p354: AF503511; F) G1, AF110391. The following accession numbers have been used for phylogenetic analysis of human isolates: AB385844–AB385848, AB385850–AB385852, DQ200279, DC200282–DQ200284, DQ200287, DQ200289, DQ200292, DQ200293. Accession numbers of animal and environmental samples are those in Figure 1.

Dutch environmental and animal HEV sequences obtained during 2004–2006 were compared with HEV ORF2 sequences from a study on Dutch hepatitis E patients without a travel history who received a diagnosis during 2004–2006 (Figure 2) (*19*). Nucleotide identities between environmental isolates and between human and environmental isolates were similar, as shown in Table 2. These results suggest that variability in HEV strains circulating in Dutch pigs, wild boar, and surface water is similar to variability in strains circulating in humans.

Human sequences grouped within the same 4 HEV genotype 3 clusters, with most of the sequences clustering within Dutch cluster 3c. A relatively high percentage of the sequences in cluster 3c is of human origin (43%). In this cluster, 1 human sequence was 100% identical with a porcine HEV sequence. For confirmation of homology, ORF1 RT-PCRs were performed, but only the pig sample yielded an ORF1 sequence. No direct geographic evidence could be established for an association between the patient and the pig farm; the patient who contracted the HEV infection in 2005 lived in the northern region of the Netherlands, but the pig farm was located in the eastern region. Comparison of postal codes of other Dutch patients without a recent travel history with postal codes of environmental sample locations did not show any geographic clustering.

In cluster 3f, only 1 (5%) of 21 sequences identified during 2004–2006 was of human origin. Comparison with HEV sequences derived from human serum samples showed 2 close homologies, 1 between sequences from a hepatitis E patient in 2005 and a pig (96.6%) and 1 between sequences from a patient in 2006 and a surface water sample in 2005 (97.3%).

## Discussion

Evidence was obtained for the presence of HEV in food, water, and animals in the Netherlands. Although 4 genotypes of HEV are known, 3 of which have been detected in pigs, each HEV ORF2 fragment sequenced in our study was identified as genotype 3. The highest prevalence (53%) was found on pig farms housing pigs 5–27 weeks of age, which is consistent with studies reporting that most HEV infections in pigs occurred between 2 and 4 months of age (*6*,*29*). However, our study on pigs of 6 months of age, the approximate age at slaughter, showed that HEV RNA was present in 14% of the pig feces samples, which corresponds to 30% of the pig farms. HEV RNA was detected in 6% of commercial pig livers, which indicated that pigs may still be a source of HEV during slaughter. This finding suggests that swine veterinarians (*30*,*31*) and other professionals, such as sewage workers (*32*), slaughterhouse workers, and butchers, who have close contact with pigs or pig products, may be exposed to the virus by working with pigs. This suggestion is supported by the fact that we detected HEV RNA in wastewater resulting from rinsing pig intestines during slaughter (S.A. Rutjes, unpub. data).

The percentage of HEV RNA–positive commercial livers was approximately half that of HEV-positive feces at the time of slaughter, which may be caused by metabolic degradation of the virus in liver tissue or by frequent freezing of livers before they are sold in supermarkets or butcher shops, which may reduce the amount of viral RNA. Alternatively, differences in HEV prevalence detected in livers and that detected in feces may be caused by differences in assay sensitivity or different distribution patterns in the 2 sample matrices. In 2 recent studies, contaminating virus in pig livers was shown to be infectious, but the risk for infection by consumption of properly cooked pig livers was extremely small (*33*,*34*).

Rodents have been suggested to be a reservoir for HEV on the basis of high seroprevalences (<73%) (*11*,*35*). We used RT-PCR to examine whether muskrats are a potential reservoir for HEV. Although no RNA was detected in muskrat fecal samples, muskrats cannot be excluded as a reservoir because of high concentrations of RT-PCR inhibitors in feces, which implies that false-negative results may have been obtained. Alternatively, HEV strains present in muskrats may not have been detected by the HEV RT-PCR used in the current study.

In addition to animal sources, HEV genotype 3 was detected in 17% of samples studied from the Meuse River. Several studies have shown that HEV originating from pigs and humans is consistently present in sewage water (*36*,*37*), which implies that surface waters may be contaminated by sewage overflows or discharge of insufficiently treated sewage water. The Meuse River runs from France through Belgium into the Netherlands and is used for recreational purposes and drinking water. Thus, water from this river is a potential source for exposure to HEV.

HEV concentrations ranging from 10^3^ PDU/g feces to 10^6^ PDU/g feces and from 0.002 PDU/mL to 0.1 PDU/mL were detected in surface water. Although these are results for genome copies and not infectious viruses, it may be concluded that in the sources examined in this study, pig feces contained the highest numbers of (infectious) virus. To confirm this hypothesis, samples should be tested for infectious virus by in vivo infection experiments (*28*) or cell culture (*38*,*39*).

In 1998–1999, HEV sequences detected in Dutch pigs grouped within clusters 3a, 3c, and 3f (*25*). In the current study, strains of an additional fourth cluster of HEV, genotype 3e, were detected in pig and surface water samples and in 2 patients whose illnesses had been diagnosed in 2005 and 2006. This result indicates that although this 3e variant was not previously detected in the Netherlands, it is now present in various sources and may have emerged a few years ago. Cluster 3c comprises 35% of animal and environmental HEV sequences and 75% of human HEV sequences and is unique to the Netherlands (*20*). This geographic clustering of genotype 3 strains has been reported in several countries (*3*). Sequences of subtypes 3e and 3f have also been detected in other European countries and Japan, whereas subtype 3a sequences have been detected mainly in the United States, Japan, and South Korea, which suggests that these subtypes may have been introduced in the Netherlands by travelers or commercial trade involving HEV-infected pigs. One genotype 3 variant detected on 1 pig farm was unrelated to variants in available databases or any human strain so far detected in the Netherlands.

The percentage of human sequences within the 2 largest clusters (3c and 3f) showed large differences (43% vs. 5%). This finding suggests that HEV 3c strains may be more pathogenic to humans, more stable in the environment, or are shed in higher numbers. Strains of feline calicivirus with different pathogenicities obtained from distinct outbreaks did not show conserved changes in virus genomes. Nevertheless, strains with higher pathogenicity infected tissue culture cells more efficiently and showed earlier cytopathic effects (*40*). To determine whether such differences in pathogenicity are also present between 3c and 3f HEV variants, a cell culture assay for HEV is needed. Despite increasing knowledge of replication and packaging of HEV in somatic cells, an efficient cell culture method is currently not available (*38*,*39*).

Of 46 animal and environmental HEV sequences, 2 strains were 100% identical on a fragment of the 148-nt ORF2, encoding the viral capsid protein. Of 16 human isolates, 2 strains were 100% identical. One HEV sequence of 148 nt of ORF2 from a patient whose condition was diagnosed in 2005 was identical to a porcine HEV strain detected in the same year. The fact that of the 46 animal and environmental HEV sequences only 2 sequences were identical indicates that sequence variability in this short fragment is high, which is suggestive of a high mutation rate in this part of the genome. Conversely, variation within herds appeared to be low (S.A. Rutjes, unpub. data) (*15*), which argues against a high mutation rate. To better understand the role of similarities of ≈100% between mutual environmental HEV strains and human strains, mutation rates of HEV in individual pigs should be studied by longitudinal follow up.

In this study, several sources of HEV have been identified that are suggestive for risk factors such as contact with pigs or wild boars or their food products, as well as consumption of those products in undercooked conditions. To reduce exposure and infection by introduction of efficient intervention measures, transmission routes have to be identified. Furthermore, different distributions of human HEV sequences between the 2 largest clusters (3c and 3f) suggest that the route of exposure and the virus subtype will play a role in HEV infection and disease in humans.
